# Narciclasine induces autophagy-mediated apoptosis in gastric cancer cells through the Akt/mTOR signaling pathway

**DOI:** 10.1186/s40360-021-00537-3

**Published:** 2021-11-09

**Authors:** Yunfeng Yuan, Xue He, Xiang Li, Yan Liu, Yueliang Tang, Huiming Deng, Xinyuan Shi

**Affiliations:** 1 Department of Hepatobiliary Surgery, Chongqing Three Gorges Central Hospital, Chongqing University Three Gorges Hospital, No.165 Xincheng Road, Chongqing, 404000 China; 2 Department of General Surgery, Zengcheng District People’s Hospital of Guangzhou, No.1 Guangming East Road, Guangzhou, 511300 China; 3 Department of Gastroenterology, Taizhou First People’s Hospital, No. 218 Hengjie Road, Taizhou, 318020 China

**Keywords:** Narciclasine, Gastric cancer, Autophagy, Apoptosis, Akt/mTOR pathway

## Abstract

**Background:**

Gastric cancer is a common gastrointestinal cancer and currently has the third-highest mortality rate. Research shows that the natural compound narciclasine has a variety of biological activities. The present study aimed to investigate the effect of narciclasine on gastric cancer cells and its molecular mechanisms and determine whether this compound could be a novel therapy for gastric cancer.

**Methods:**

MTT and clone assays were employed to detect the proliferation of gastric cancer cells. The cell apoptosis was detected by flow cytometry. The formation of autophagosomes and autophagosomal lysosomes was observed by transmission electron microscopy and laser confocal scanning microscopy. Western blotting was used to detect the expression of apoptosis, autophagy and Akt/mTOR pathway-related proteins.

**Results:**

In this study, we found that narciclasine could inhibit the proliferation of gastric cancer cells and promote apoptosis in gastric cancer cells. Further experiments showed that narciclasine promoted the levels of autophagy proteins LC3-II, Atg-5 and Beclin-1, reduced the expression of the autophagy transporter p62, and increased autophagic flux. By using the autophagy inhibitors 3-MA and CQ, it was shown that narciclasine could induce autophagy-mediated apoptosis in gastric cancer cells. Finally, we found that narciclasine had no significant effects on the total content of Akt and mTOR in gastric cancer cells, and it involved autophagy in gastric cancer cells by reducing the phosphorylation level of p-Akt and p-mTOR.

**Conclusions:**

Narciclasine can induce autophagy-dependent apoptosis in gastric cancer cells by inhibiting the phosphorylation level of Akt/mTOR and thus reduce the proliferation of gastric cancer cells.

## Background

Gastric cancer is a common gastrointestinal tract cancer. According to global cancer data in 2018, the number of people with gastric cancer was about 1.03 million, with 0.78 million reported deaths, ranking it the second most common among 36 types of cancer. Compared with 2012, both new cases and mortality have increased significantly [1, 2]. Although certain progress has been made in surgical treatment, radiotherapy and chemotherapy of gastric cancer in the past 10 years, the overall mortality rate has not been significantly reduced. The main reasons are the low surgical resection rate, the significant side effects of chemotherapy and the high recurrence rate after chemotherapy. Therefore, it is urgent to find a new way to treat gastric cancer.

Cell autophagy is a process in which cells degrade the damaged organelles and macromolecular substances using lysosomes under the control of autophagy-related genes (Atg), which is a self-protection mechanism of cells [3, 4]. Under physiological conditions, autophagy can provide energy to the body and maintain intracellular homeostasis by degrading aging proteins and damaged organelles in cells [5]. Autophagy is also closely related to the occurrence and development of tumors. On the one hand, autophagy can help tumor cells better adapt to external stress and contribute to cancer cell proliferation, invasion, and drug resistance [6, 7]. On the other hand, autophagic death of cancer cells can be induced when the autophagy activation increases or continues to occur [8, 9]. Therefore, it is hopeful that the development of drugs that promote autophagy of gastric cancer cells is one potentially important mechanism to improve the survival rate of gastric cancer patients.

Narciclasine is a plant growth inhibitor isolated from the mucus secreted by narcissus bulbs. In 1967, Ceriotti et al. isolated narciclasine for the first time and in 1997 Rigby et al. completed the first organic synthesis of this compound [10, 11]. Many studies in recent years have shown that narciclasine has various biological activities such as anti-inflammatory [12], angiogenesis inhibitory [13], antiviral [14], and anti-tumor effects [15], and so on. Therefore, the present study aims to investigate the effect of narciclasine on gastric cancer cells and its molecular mechanisms. We selected moderately and poorly differentiated BGC-823 and SGC-7901 gastric cancer cells, highly differentiated MGC-803 and MKN28 cells, and human gastric mucosal epithelial GES-1 cells for investigation.

In the present study, we found that narciclasine could inhibit the proliferation of gastric cancer cells by stimulating autophagy. Its potential mechanism is to inhibit phosphorylation of the Akt/mTOR signaling pathway and activate autophagy, thus promoting apoptosis of gastric cancer cells.

## Materials and methods

### Reagents and cell culture

**Fig. 1:**
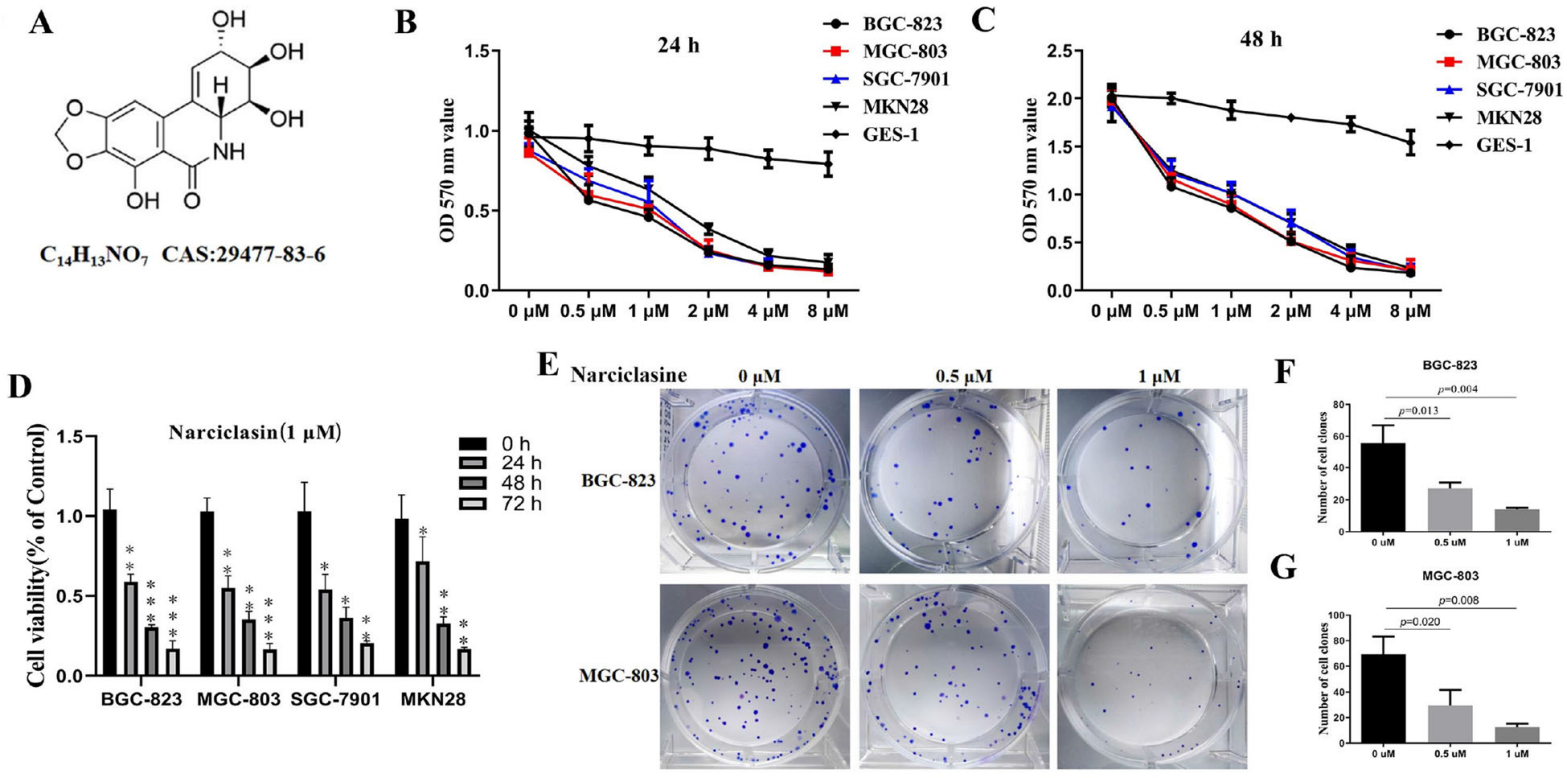
Narciclasine inhibits the proliferation of gastric cancer cells. A, Chemical structure of narciclasine. B-D, Gastric cancer cells BGC-823, SGC-7901, MKN28, MGC-803 and gastric mucosal cells GES-1 were treated with indicated concentrations of narciclasine and the same concentration of narciclasine at different time, MTT assay was used to measure cell viability, Data are shown as mean ± SD. Compared with the control group, *P < 0.05; **P < 0.01;***P < 0.001. E-G, Effects 0.5 μM and 1 μM narciclasine on colony formation of gastric cancer cells BGC-823 and SGC-7901

Narciclasine (Fig. 1a) was purchased from Chengdu Herbpurify CO., LTD (Chengdu, China), with a purity ≥99.9%. The human gastric cancer cell lines BGC-823, MGC-803, GES-1, MKN28 and SGC-7901 were purchased from the Institute of Biochemistry and Cell Biology at the Chinese Academy of Sciences (Shanghai, China); Roswell Park Memorial Institute 1640 (RPMI 1640), fetal bovine serum (FBS), penicillin and streptomycin from Gibco Life Technologies (NY, US); chloroquine (CQ) and 3-methyladenine (3-MA) from Selleck Chemicals (Texas, US); insulin from Sigma-Aldrich (CA, US); and AktiVIII from Macklin Inc. (Beijing, China). Human gastric cancer cells were cultured in RPMI 1640 medium (containing 10% FBS and 2.5% penicillin-streptomycin) and then cultured in a constant temperature incubator at 37 °C with 5% CO_2_ saturation humidity.

### MTT assay and colony formation assay

Gastric cancer MGC-803, BGC-823, SGC-7901, MKN28 and GES-1 cells in the logarithmic growth phase were seeded in 96-well plates with 5 × 10^3^ cells per well and 5 replicates in each group. The cells were treated with narciclasine. After 24 h or 48 h, 10 μL of MTT (C0009, Beyotime Biotechnology, Shanghai, China) solution (5 mg/mL) was added to each well. After being placed in an incubator for 4 h, the MTT-containing culture solution was aspirated and discarded 150 μL of DMSO was added to each well, which was shaken gently for 5–10 min on a mechanical shaker. The OD value of each well was read at 570 nm on a microplate reader (SpectraMaxM4, MD Company, US). Based on the IC_50_ values of gastric cancer cells during 24 h, we chose the gastric cancer BGC-823 and MGC-803 cells for further experiments.

200 gastric cancer MGC-803 and BGC-823 cells were seeded onto 12-well plates and narciclasine with the final concentrations of 0.5 μM and 1 μM were added to the culture medium, respectively. An equal amount of drug-free culture solution was added to the control group and then was incubated for 14 days. Colonies were fixed in 4% paraformaldehyde for 20 min, washed three times with PBS, and stained with 0.1% crystal violet (Sigma-Aldrich, CA, US) for 30 min, and then washed three times with PBS again. Visible colonies were photographed by microscope and the number of colonies was counted by Image J software.

### Flow cytometry assay

The gastric cancer cells were harvested and resuspended in 500 μL of 1 × binding buffer. 5 μL annexin V-FITC was added to the cell suspension first, and then 10 μL PI (Annexin V-FITC Detection Kit, Beyotime Biotechnology, Shanghai, China) was added. The cells were evenly mixed and then incubated in a refrigerator at 4 °C for 10 min in the dark. Cells were detected by flow cytometry (Accuri C6, Becton-Dickinson, US).

### Transmission electron microscopy

The gastric cancer cells (2 × 10^5^ - 1 × 10^6^) were harvested after being treated with narciclasine for 24 h. The cells fixed were with 2.5% glutaraldehyde for 4 h in a volume 20 times larger than the sample volume and then rinsed with 0.1 MPBS. After that, they were fixed with 1% osmic acid for 1 h, rinsed twice in double-distilled H_2_O for 10–15 min and finally fixed/stained with 2% uranium acetate for 30 min. The cells then underwent gradient dehydration with alcohol, pure acetone + (1,1) penetrating embedding agent treatment, temperature gradient polymerization in an oven, ultra-thin sectioning using a microtome, and uranium acetate-lead citrate staining, before finally being examined using a transmission electron microscope (Philips Co. Ltd., Netherlands).

### Laser confocal scanning microscope observation

Cells were infected (MOI = 300) with mRFP-GFP-tagged LC3 (tfLC3) (Hanbio Biotechnology Co. Ltd., Shanghai, China) adenovirus and treated with narciclasine for 24 h, and then fixed in formaldehyde at room temperature for 20 min. 5 μL of glycerol-PBS was added dropwise to each glass slide, which was then immersed in the cell culture medium for fixation. Any fluorescence changes of GFP and RFP in cells were observed using a laser confocal scanning microscope (Olympus Co., Ltd., Japan), and the number of autolysosomes and autophagosome were calculated.

### Western blotting

Gastric cancer cells were lysed with RAPI lysate (Beyotime Biotechnology, Shanghai, China) and centrifuged at 4 °C for 30 min at 14,000 rpm to concentrate and collect the proteins. The concentration of proteins in each sample was determined using a BCA protein assay kit (Beyotime Biotechnology, Shanghai, China). After electrophoresis with 10% SDS-PAGE, the proteins were transferred to a PVDF membrane (Millipore, MA, US), blocked with 5% skimmed milk for 2 h, and then washed 3 times in TBST buffered saline. The PVDF membrane was cut according to protein molecular weight and experimental grouping, and they were then incubated with Bax, Bcl-2, cleaved-PARP, cleaved-caspase-3, 8, 9, cyto-c, LC3-II, Beclin1, p62, Akt, p-Akt, mTOR and p-mTOR (1:1000, Abcam, Cambridge, MA, USA) primary antibodies overnight at 4 °C. After being washed three times with TBST buffer, the membrane was incubated in IgG antibodies 1:2000 dilution (MultiSciences, Shanghai, China) at room temperature for 1 h, and then washed three times with TBST buffer salt. An ECL chemiluminescence kit (Beyotime Biotechnology, Shanghai, China) was used for color development in a dark room, and then gel images were collected and analyzed. The corresponding gray value of each image strip was analyzed using Quantity One software.

### Statistical analysis

All values were presented as mean ± standard deviation (SD). GraphPad Prism ver. 6.0 software (La Jolla, CA, US) was used for all statistical analyses. One-way ANOVA was applied to analyze the differences among three or more groups, and the differences between two groups were analyzed with Student’s t test. A *P*-value < 0.05 was considered statistically significant.

## Results

### Narciclasine inhibits proliferation and promotes apoptosis of gastric cancer cells

**Fig. 2:**
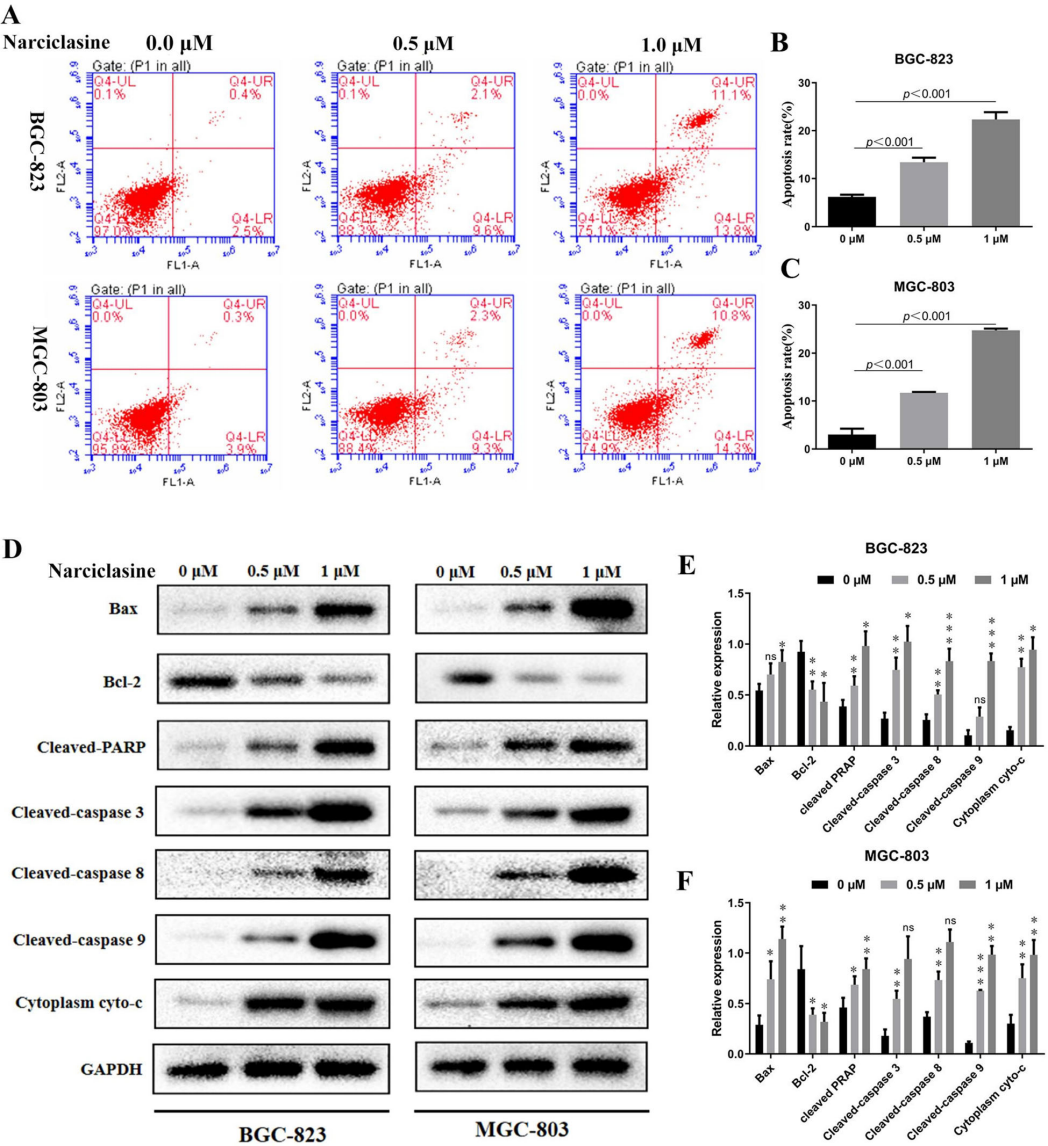
Narciclasine promotes apoptosis of gastric cancer cells. A-C, The apoptosis rate of gastric cancer cells was detected by flow cytometry after treatment with narciclasine for 24 h. D-F, Western blotting was used to detect the effect of narciclasine on the expression of the apoptotic proteins Bcl-2, Bax, cleaved-PARP, cleaved-caspase-3, 8, 9 and cyto-c in gastric cancer cells after 24 h. Data are shown as mean ± SD. Compared with the control group, *P < 0.05; **P < 0.01;***P < 0.001. ns: no significance

In order to investigate the inhibitory effect of narciclasine on the proliferation of gastric cancer cells, we used the MTT assay to detect the viability of gastric cancer and gastric mucosal cells after treatment with different concentrations of narciclasine and the same concentration of narciclasine at different time. The results showed that narciclasine significantly inhibited the proliferation of gastric cancer cells BGC-823, SGC-7901, MGC-803 and MKN28 with a dose-dependent and time-dependent manner, but it had a weak toxic effect on gastric mucosal cells GES-1 (Fig. 1b-d). Similarly, our cell colony investigations showed that the number of cell colonies in the narciclasine group was significantly less than in the control group (Fig. 1e-g). Flow cytometry was used to further clarify whether the inhibitory effect of narciclasine on the proliferation of gastric cancer cells was related to apoptosis, as shown in Fig. 2a-c. The apoptosis rate of gastric cancer cells in the treatment groups exposed to 0.5 μM and 1 μM narciclasine was significantly increased. This was further supported by the increased expression of Bax, cleaved-PARP, cleaved-caspase-3, 8, 9, and cyto-c, while the Bcl-2 expression in the narciclasine group was significantly decreased (Fig. 2d-f). These results indicated that narciclasine could inhibit the proliferation and promote the apoptosis of gastric cancer cells.

### Narciclasine promotes autophagy in gastric cancer cells

**Fig. 3:**
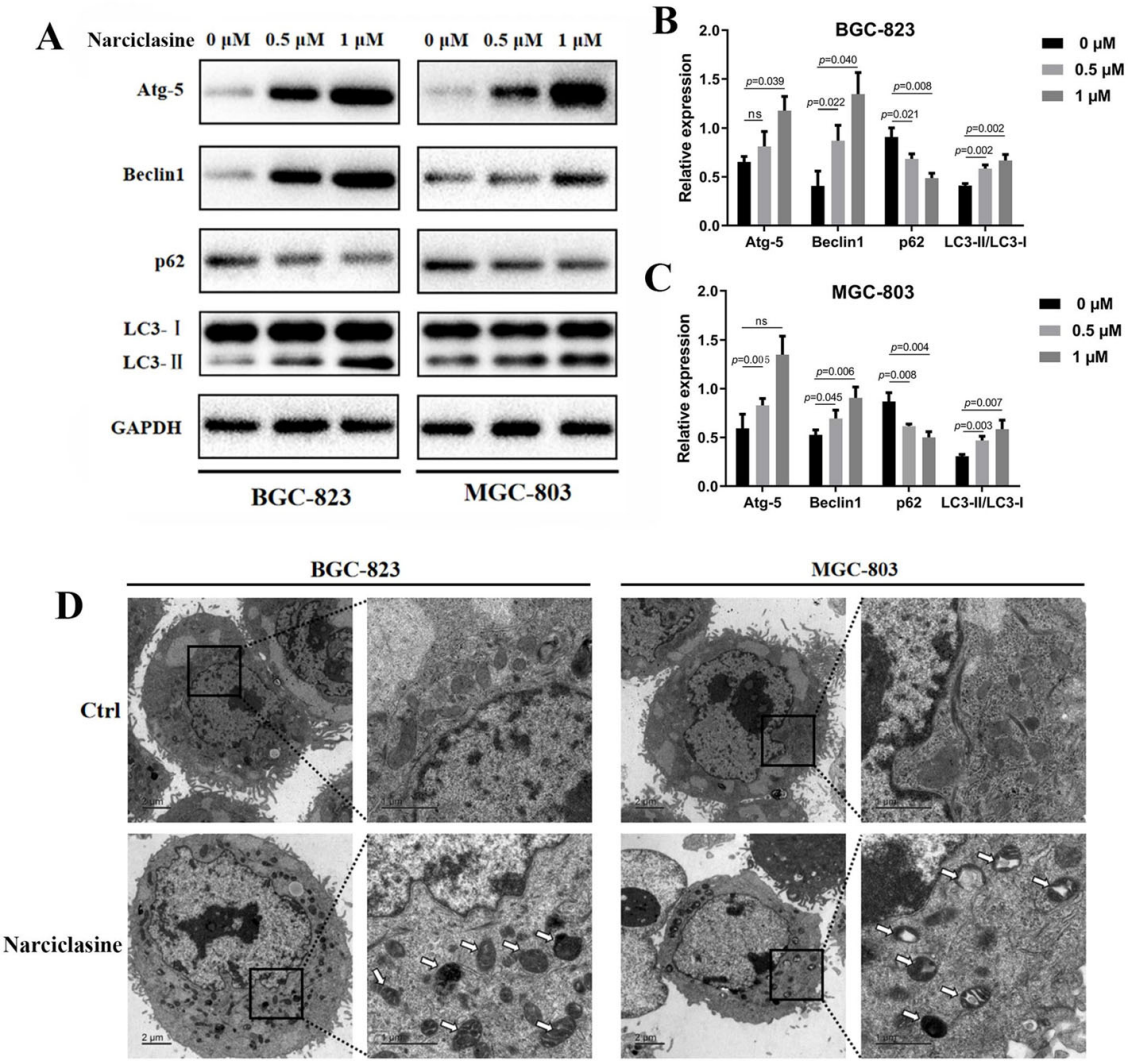
Narciclasine mediates autophagy in gastric cancer cells. A-C, Western blotting was used to detect the effect of narciclasine on the expression of autophagy proteins LC3-II, Atg-5, p62 and Beclin1 in gastric cancer cells after 24 h. Data are shown as mean ± SD. ns: no significance. D, Transmission electron microscopy was used to observe the formation of autophagosomes in gastric cancer cells treated with narciclasine (0.5 μM) for 24 h

**Fig. 4:**
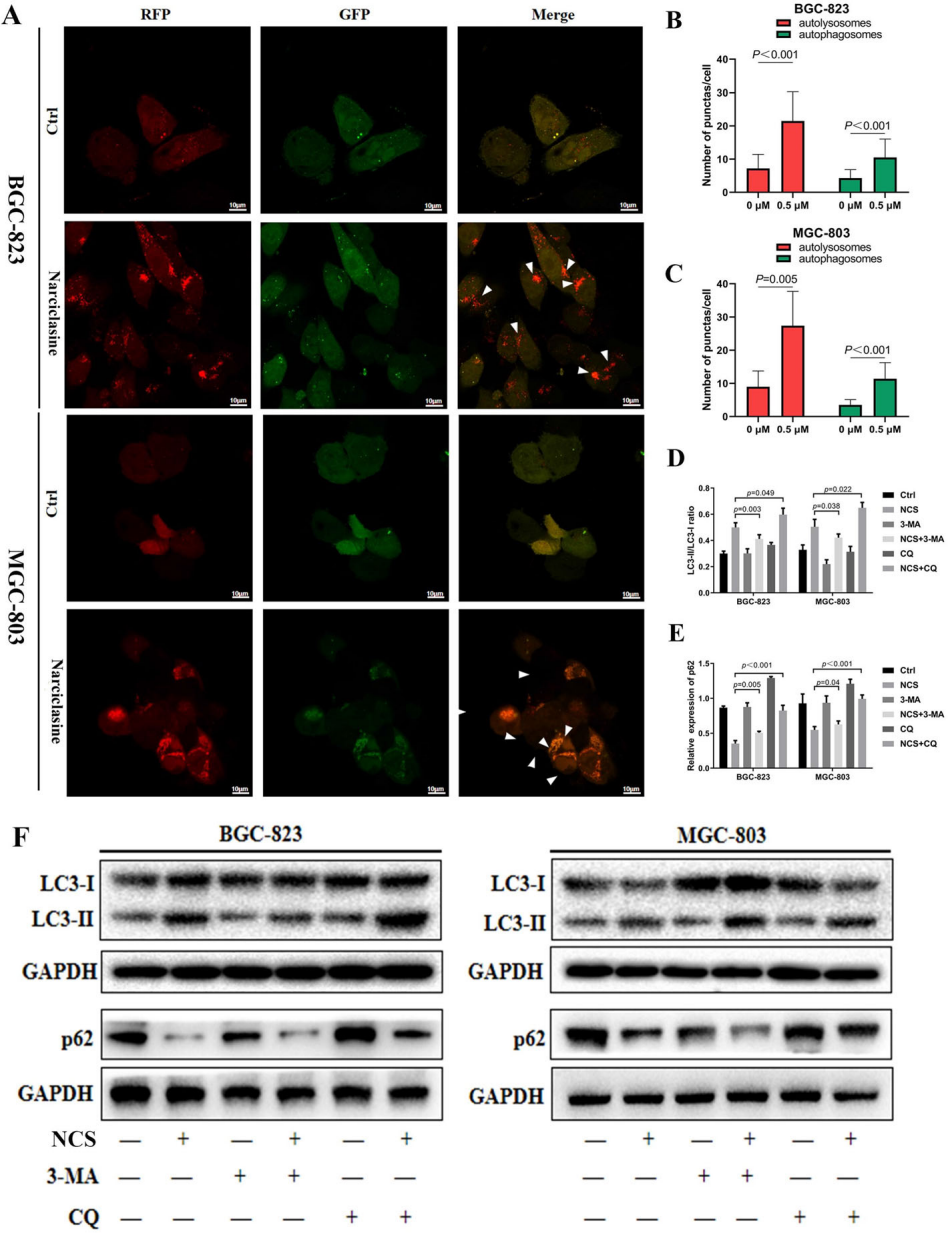
Narciclasine promotes autophagy of gastric cancer cells. A-C, Laser confocal scanning microscopy was used to observe the formation of autolysosomes in gastric cancer cells treated with narciclasine (0.5 μM) for 24 h. D-F, Western blotting was used to detect the effect of narciclasine combined with autophagy inhibitor 3-MA (5 mM) and CQ (2.5 μM) on LC3-II and p62 protein after treatment of gastric cancer cells for 24 h. Data are shown as mean ± SD. NCS: narciclasine; 3-MA: 3-methyladenine; CQ: chloroquine

In some situations, persistently activated or reinforced autophagy can lead to apoptosis of cancer cells [16], so we explored whether narciclasine could induce autophagy in gastric cancer cells. Firstly, we measured the expression of Atg-5, p62, Beclin1 and the conversion of LC3-I to lipidated LC3-II. As shown in Fig. 3a-c, the expression of LC3-II, Atg-5 and Beclin1 in the narciclasine group was significantly increased, while the expression of the autophagosome transporter p62 was decreased compared to the control group. Similarly, using electron microscope, we observed a significant accumulation of autophagosomes or autolysosomes in narciclasine-treated cells but not in those of the control group (Fig. 3d). In order to further prove that narciclasine promotes autophagy flux, thus, we used mRFP-GFP-tagged LC3 to track the accumulation of autophagosomes or autolysosomes [17]. The results revealed that the number of autophagosomes and autolysosomes in the gastric cancer cells in the narciclasine-treated group was significantly greater than that in the control group (Fig. 4a-c), which suggested that autophagosomes could successfully bind to lysosomes instead of being blocked. We used 3-MA (an autophagosome formation inhibitor) and CQ (autophagosome lysosomal binding inhibitor) to further demonstrate that narciclasine could induce an increase in the number of autophagosomes of gastric cancer cells and promote autolysosomes formation (i.e., promote autophagy). The results are shown in Fig. 4d-f. Narciclasine combined treatment with 3-MA markedly reduced the LC3-II conversion compared with narciclasine-treated gastric cancer cells. The combinatorial treatment with CQ resulted in the accumulation of LC3-II indicating that 3-MA and CQ could reverse part of the role of narciclasine in promoting autophagy. In addition, we also found that 3-MA and CQ could reverse the expression of p62, which indicated that 3-MA and CQ could reduce the role of narciclasine in promoting the dissolution of p62 in lysosomes. Thus, narciclasine can enhance autophagy in gastric cancer cells.

### Narciclasine induces apoptosis by enhancing autophagy of gastric cancer cells

**Fig. 5:**
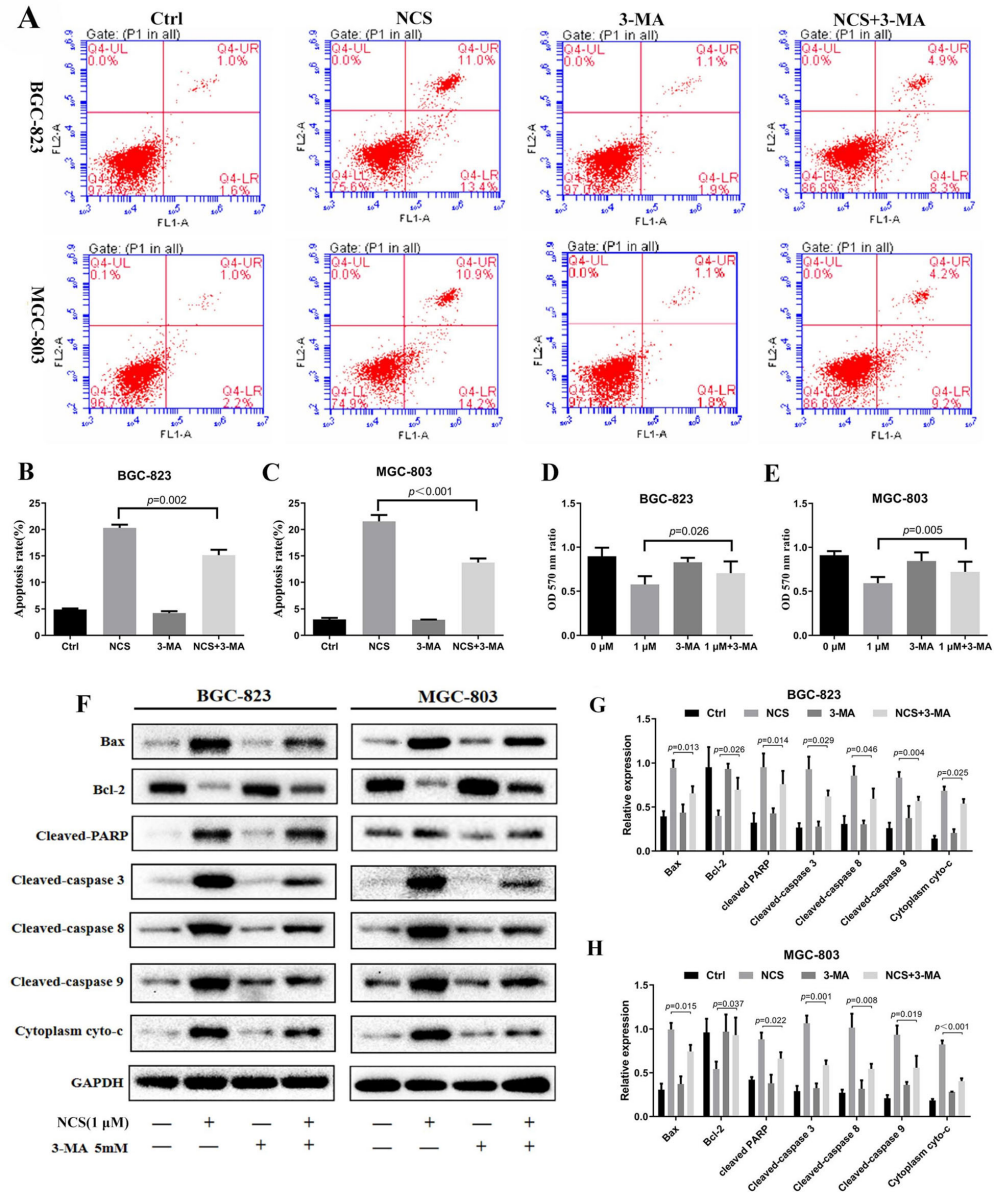
Narciclasine mediates autophagy-dependent apoptosis of gastric cancer cells. A-C, Flow cytometry was used to detect the apoptosis rate of gastric cancer cells treated with narciclasine combined with the autophagy inhibitor 3-MA (5 mM) for 24 h. D-E, The MMT assay was used to detect the cell viability of gastric cancer cells treated with narciclasine combined with the autophagy inhibitor 3-MA (5 mM) for 24 h. F-H, Western blotting was used to detect the effects of narciclasine combined with the autophagy inhibitor 3-MA (5 mM) on the expression of apoptotic proteins Bcl-2, Bax, cleaved-PARP, cleaved-caspase-3, 8, 9 and cyto-c in gastric cancer cells after 24 h. Data are shown as mean ± SD. NCS: narciclasine; 3-MA: 3-methyladenine

Although autophagy and apoptosis are two forms of programmed cell death, they can co-exist or promote each other when driven by specific external stimuli [18]. Next, we explored whether the apoptosis promoted by narciclasine was related to enhanced autophagy effects. We first inhibited narciclasine-induced autophagy by autophagic inhibitors (3-MA) and observed the effects on apoptosis in gastric cancer cells. The results are presented in Fig. 5a-c, which shows that 3-MA could reduce apoptosis caused by narciclasine. Consistently, the 3-MA greatly rescued the narciclasine-mediated up-regulation of Bax, cleaved-PARP, cleaved-caspase-3, 8, 9 and cyto-c, and promoted the expression of Bcl-2(Fig. 5f-h). Similarly, the results of MTT experiments revealed that the activity of gastric cancer cells was further strengthened when combined with 3-MA treatment (Fig. 5d-e), suggesting that autophagy inhibitors can reduce the pro-apoptosis and anti-proliferation effects of narciclasine on gastric cancer cells. This in turn proves that narciclasine promotes gastric cancer apoptosis and inhibits proliferation by enhancing autophagy.

### Narciclasine promotes autophagy-induced apoptosis in gastric cancer cells through Akt/mTOR

**Fig. 6:**
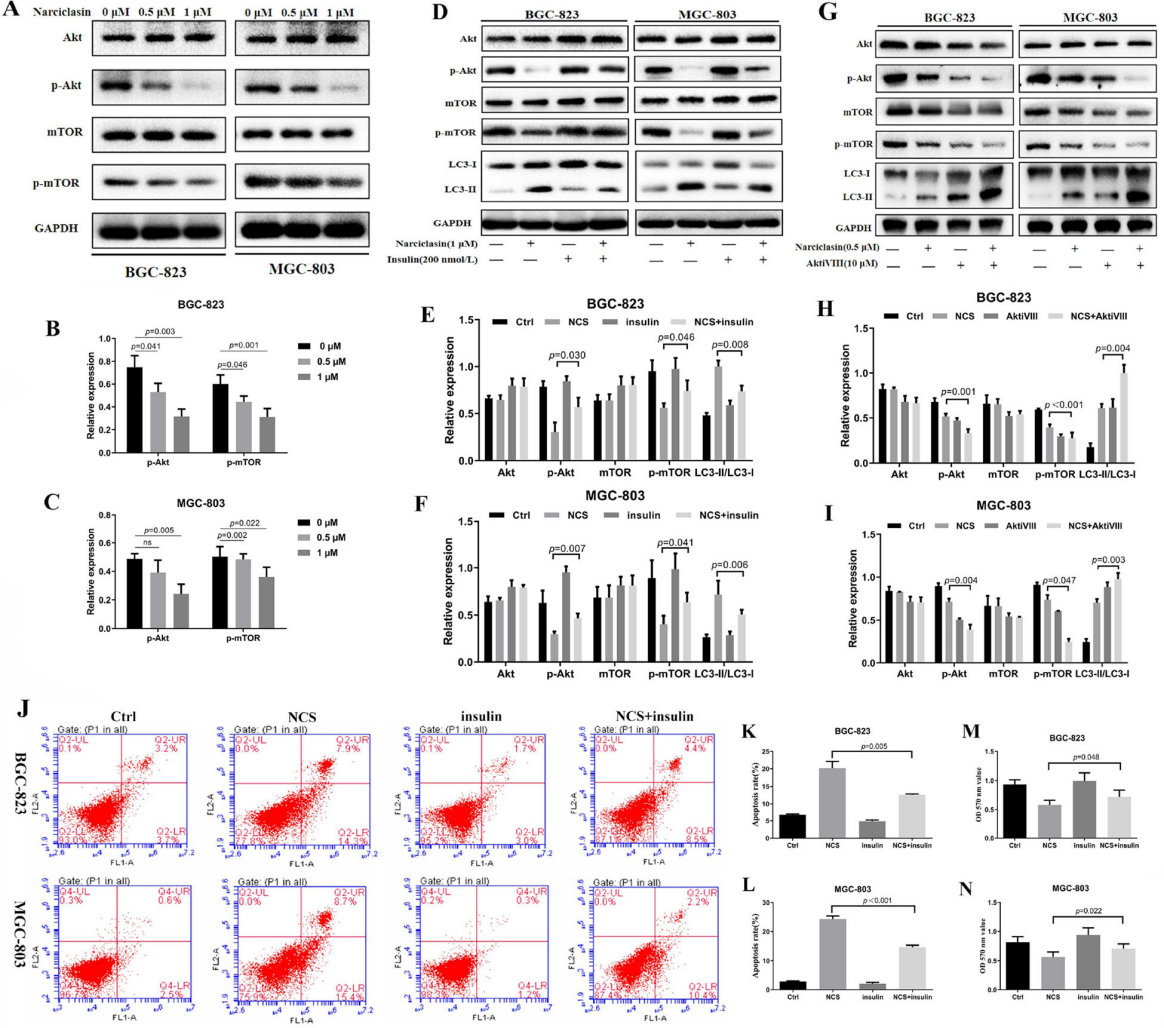
Narciclasine mediates autophagy-dependent apoptosis of gastric cancer cells by inactivation of the Akt/mTOR pathway. A-C, Western blotting was used to detect the effect of narciclasine on total Akt and mTOR, p-Akt and p-mTOR of gastric cancer cells. D-I, Western blotting was employed to detect the effects of narciclasine combined with the Akt agonist insulin (200 nmol/L) and Akt inhibitor AktiVIII (10 μM) on Akt, mTOR, p-Akt, p-mTOR, and LC3-II protein after treatment of gastric cancer cells for 24 h. J-L, The apoptosis rate of gastric cancer cells treated with narciclasine and Akt agonist insulin (200 nmol/L) for 24 h was detected by flow cytometry. M-N, MMT assay was used to detect the 24-h cell viability of gastric cancer cells treated with narciclasine combined with the Akt agonist insulin (200 nmol/L). Data are shown as mean ± SD. NCS: narciclasine.ns: no significance

Previous studies have shown that Akt/mTOR signaling pathway is closely related to autophagy [19, 20], so we investigated whether narciclasine affected Akt/mTOR signaling pathway in gastric cancer cells. We found that narciclasine significantly inhibited levels of p-AKT and p-mTOR, but not the total protein levels of Akt and mTOR (Fig. 6a-c). To further demonstrate the role of Akt/mTOR dephosphorylation in narciclasine-induced autophagy, we treated gastric cancer cells with insulin (Akt activator) to reactivate Akt/mTOR signaling pathway. The results showed that the insulin rescued narciclasine-mediated downregulation of p-AKT and p-mTOR, and decreased the expression of LC3-II (Fig. 6d-f). Interestingly, when we used AKTiVIII (Akt/mTOR inhibitors) combined with narciclasine to treat gastric cancer cells, the conversion level of LC3-II was significantly increased compared with the narciclasine group, while the expression levels of p-AKT and p-mTOR decreased (Fig. 6g-i). In addition, our results revealed that the apoptosis rate of gastric cancer cells in the narciclasine combined with the insulin group was lower than that in the narciclasine group, and insulin could reduce the effects of narciclasine on inhibiting the proliferation of gastric cancer cells (Fig. 6j-n). In summary, these results strongly indicate that narciclasine-induced autophagy is associated with inhibiting the phosphorylation level of Akt/mTOR pathway in gastric cancer cells, thereby promoting apoptosis in gastric cancer cells and inhibiting cell proliferation.

## Discussion

It is well known that many natural compounds have potent anti-cancer effects, such as vincristine, irinotecan, etoposide and paclitaxel, etc., and they often have the characteristics of low toxicity, high efficiency, low cost and wide sources [21–23]. Narciclasine is abundant in fresh bulbs of natural plants such as *Lycoris radiata*, tiger ear, water ghost coke, snowflake genus, summer snowflake and yellow *Lycoris radiata* [14]. Previous studies have shown that narciclasine can act highly and selectively on glioblastoma multiforme and inhibit the growth of Hela tumor cells [24, 25]. The present study results indicated that narciclasine at a low concentration could significantly inhibit the proliferation of BGC-823, MGC-803, MKN28 and SGC-7901 cells and inhibit colony formation of gastric cancer BGC-823 and MGC-803 cells. According to previous research, narciclasine is highly selective for cancer cells, and its toxic effect on normal fibroblasts is 250 times lower than that of cancer cells; therefore, it is unlikely to cause apoptosis of normal fibroblasts at therapeutic doses [26]. It is noteworthy that we found the toxic effect of narciclasine was weak on gastric mucosal cells GES-1, but highly selective toxic on gastric cancer cells, indicating that narciclasine has great potential to be an anti-gastric cancer drug.

Research has shown that in human breast cancer MCF-7 cells and PC-3 prostate cells, narciclasine can activate Caspase-8 and Caspase-10 receptor pathways and induce apoptosis of tumor cells [27]. In the present study, we also found that narciclasine could promote the apoptosis of gastric cancer cells, inhibit the expression of Bcl-2 and increase the expression of Bax, cleaved-PARP and cleaved-3,8,9, suggesting that narciclasine may activate the apoptosis pathway in gastric cancer cells. In addition, we confirmed that narciclasine could enhance the autophagy of gastric cancer cells, for example, using autophagy inhibitors.

Several relationships co-exist between autophagy and apoptosis: autophagy and apoptosis work together to promote cell death, with autophagy and apoptosis antagonizing each other; autophagy provides cells with the energy necessary for survival and inhibits their apoptosis; and autophagy promotes the transformation of cells to apoptosis [28, 29]. Cao et al .[30] found that narciclasine could inhibit the proliferation of breast cancer cells by mediating autophagy-dependent apoptosis via AMPK-ULK1 signaling. In our study, we used 3-MA to inhibit autophagy and found that narciclasine induced a decrease in apoptosis of gastric cancer cells. Therefore, it could be speculated that narciclasine promotes the apoptosis of gastric cancer cells by activating autophagy. The interaction between Beclin1 and Bcl-2 is one of the common regulatory modes of autophagy and apoptosis [31]. Because of the BH3 domain on Beclin1, apoptotic factors such as Bcl-2 and Bcl-xl can affect autophagy and apoptotic activity by binding to BH 3[32]. When Beclin1 expression levels are elevated, the pro-apoptotic protein Bak/Bax, attached to Bcl-2, can be released, thus promoting apoptosis. Besides, decreased Bcl-2 expression can also lead to more Beclin1-dependent autophagy [33, 34]. Recent studies have shown that apoptosis-related proteins such as PUMA, Noxa, Nix, Bax, XIAP and Bim can also regulate autophagy. There are also reports about endogenous apoptosis regulated by autophagy proteins that control nucleation and elongation through cleavage of autophagy-related proteins by calpain and caspase [35]. Although this study confirmed that narciclasine could inhibit the expression of Bcl-2 and promote the expression of Beclin1, whether narciclasine-mediated autophagy and apoptosis of gastric cancer cells are related to the Beclin1-Bcl-2 regulatory mode needs further verification in future studies.

In 1975, Carrasco et al. found that narciclasine could effectively inhibit the biosynthesis of eukaryotic ribosome proteins and had anti-tumor effects on rabbit reticulocytes and non-cellular lines, and the mechanism of action was to bind to the 60S ribosome group to inhibit the formation of chemical bonds [36]. In addition, narciclasine can also regulate the Rho/Rho-kinase / LIM kinase cofilin signaling pathway, strengthen the activity of GTPase RhoA and reduce the formation of actin stress fibers [24]. Akt is a serine/threonine protein kinase, also known as protein kinase B. When activated, Akt will regulate many transcription factors and activate a variety of substrates, including the target proteins of rapamycin (mTOR), which stimulates cell autophagy and proliferation and inhibits apoptosis. In exploring the mechanism of narciclasine against gastric cancer, we found that narciclasine could inhibit the expression of p-Akt and p-mTOR. When activated with insulin, Akt could reduce the phosphorylation level of p-Akt and p-mTOR, and reduce the degree of autophagy and apoptosis of gastric cancer cells, suggesting that the inhibition of Akt/mTOR phosphorylation induced by narciclasine is one of its important mechanisms against gastric cancer. But the anti-tumor effect of drugs often involves multiple signaling pathways. Therefore, we will clarify the anti-tumor effects of narciclasine through multiple pathways and at multiple levels in the future. In addition, narciclasine’s anti-cancer effect and mechanisms of action in vivo also need to be verified.

## Conclusions

The results of our in vitro studies indicate that the natural compound narciclasine can mediate autophagy-dependent apoptosis of gastric cancer cells by inhibiting the phosphorylation level of Akt/mTOR. This research is expected to promote narciclasine as a novel targeted drug urgently needed to treat gastric cancer.

## Data Availability

The datasets used and/or analysed during the current study are available from the corresponding author on reasonable request.

## Abbreviations

NCS: Narciclasine
3-MA: 3-Methyladenine
CQ: Chloroquine
LC3: Light chain 3
p62: Sequestosome 1/p62
Atg-5: Autophagy-related gene 5
cyto-c: Cytochrome c
